# Axial Spinal Traction as a Potential Modulator of Cerebrospinal and Glymphatic Circulation in Neurodegenerative Diseases: A Technical Report and Biomechanical Hypothesis

**DOI:** 10.7759/cureus.99153

**Published:** 2025-12-13

**Authors:** Huan-Wei Chen

**Affiliations:** 1 Chiropractic, Private Chiropractic Practice, Vancouver, CAN

**Keywords:** alzheimer’s disease, amyotrophic lateral sclerosis (als), axial spinal traction, cerebrospinal fluid dynamics, frontotemporal dementia (ftd), glymphatic system, huntington disease, neurodegenerative diseases, parkinson’s disease, spinocerebellar ataxias

## Abstract

Impairment of glymphatic function contributes to the accumulation of metabolic and proteinaceous waste products implicated in neurodegenerative diseases such as Alzheimer’s disease, frontotemporal dementia, Parkinson’s disease, amyotrophic lateral sclerosis, Huntington’s disease, and certain spinocerebellar ataxias. Pelvis-stabilized axial spinal traction (PSAST) is a biomechanical technique designed to produce brief, controlled cranio-caudal elongation of the vertebral column and spinal dural sac, potentially generating transient pressure gradients capable of influencing cerebrospinal fluid (CSF) dynamics and glymphatic circulation.

The technique has been applied in the author’s musculoskeletal practice for more than eight years without observed persistent or treatment-related adverse effects, although such practice-based experience does not constitute a formal safety evaluation. Improved sleep quality has been the most consistently reported patient-perceived response to PSAST, a clinically notable observation given the dependence of glymphatic function on consolidated slow-wave sleep. These practice-based observations provide preliminary, hypothesis-generating support for exploring whether controlled axial elongation may modulate cerebrospinal and glymphatic physiology.

To the best of the author’s knowledge, this report presents the first peer-reviewed technical description of a reproducible, whole-axis axial spinal traction procedure with defined force parameters intended to examine potential modulation of CSF and glymphatic circulation. The report outlines the PSAST protocol and its biomechanical rationale and safety considerations and proposes its potential relevance as a noninvasive, investigational approach for conditions associated with impaired glymphatic function.

## Introduction

The glymphatic system is an astroglia-dependent perivascular clearance pathway essential for maintaining central nervous system homeostasis. It removes metabolic and neurotoxic waste products through convective exchange between cerebrospinal fluid (CSF) and interstitial fluid (ISF), a process that operates most efficiently during slow-wave (deep) sleep. Glymphatic efficiency declines with aging, driven by reductions in arterial pulsatility, impaired polarization of aquaporin-4 (AQP4) on astrocytic end feet, and structural changes that diminish CSF-ISF exchange [1-3].

Impaired glymphatic function has been increasingly implicated in the accumulation of metabolic byproducts and misfolded proteins associated with neuronal injury in many neurodegenerative disorders. Reduced clearance capacity has been linked to pathological processes in Alzheimer’s disease, frontotemporal dementia, Parkinson’s disease, amyotrophic lateral sclerosis (ALS), and Huntington’s disease and may also extend to certain spinocerebellar ataxias based on emerging imaging evidence [1-5]. Pelvis-stabilized axial spinal traction (PSAST) is a controlled cranio-caudal traction maneuver that briefly elongates the vertebral column and applies longitudinal strain to the spinal dural sac, generating transient biomechanical effects that may influence CSF dynamics. The technique is applied within physiological ranges and has demonstrated a wide apparent clinical safety margin in routine practice.

In the author’s clinical experience, improved sleep quality has been the most consistently reported effect following PSAST. Given the dependence of glymphatic function on deep, consolidated sleep [1-3], this observation is noteworthy. Furthermore, a previously reported case of probable bulbar-onset ALS demonstrated preserved bulbar and respiratory function for 21 months, now extended to 24 months on follow-up, providing preliminary, hypothesis-generating support for the possibility that controlled axial spinal elongation may modulate cerebrospinal and glymphatic circulation [6].

To the best of the author’s knowledge, no prior peer-reviewed publication has provided a technically reproducible, whole-axis axial spinal traction procedure with specified loading parameters designed to investigate potential effects on cerebrospinal or glymphatic physiology. Although the proposed mechanisms remain hypothetical, they are biomechanically grounded and provide a coherent framework for future experimental and clinical investigation.

## Technical report

Pelvis-stabilized axial spinal traction

PSAST is a practitioner-applied long-axis traction method intended to achieve controlled craniospinal elongation while minimizing practitioner exertion and maintaining biomechanical efficiency. Most chiropractic, massage, or physiotherapy tables with a central division can be adapted for PSAST by installing a pelvic stabilization belt at the midline to secure the pelvis. Additional structural reinforcement is required to prevent headward displacement of the treatment table during traction.

The patient lies supine on a stable treatment table with the pelvis secured by a cushioned restraint strap to prevent slippage and reduce localized pressure. A second webbing strap, constructed from a vehicle-seatbelt-grade material and sewn into a continuous loop approximately equal in length to the practitioner’s height, is used to support the occipital region. The strap is placed across the practitioner’s upper back, allowing traction forces to be transmitted through coordinated body mechanics rather than isolated arm effort. The practitioner positions the fingers (excluding the thumbs) beneath the occiput and places the thenar eminences over the maxilla. This hand-and-strap configuration provides smooth, symmetric, and reproducible traction while maintaining secure head support.

The traction parameters described in this section are based on the author’s long-term clinical experience and have no established precedence in published biomechanical literature. Each traction cycle consists of a gradual, controlled pull lasting approximately 2-5 seconds. The traction force is applied smoothly, increasing from baseline to the target level, typically 50-80% of body weight, over roughly two seconds rather than as a sudden or jerking motion. While the upper limit of 80% of body weight should be observed, a practical ceiling of approximately 800 N reflects the maximum load that a trained practitioner can comfortably and consistently generate in the author’s clinical experience. In practice, clinically effective forces are often lower. After reaching the target force, the applied tension may be briefly maintained for 0-3 seconds before a controlled release, with both magnitude and duration adjusted according to patient comfort, tissue resistance, and observed clinical response.

**Figure 1:**
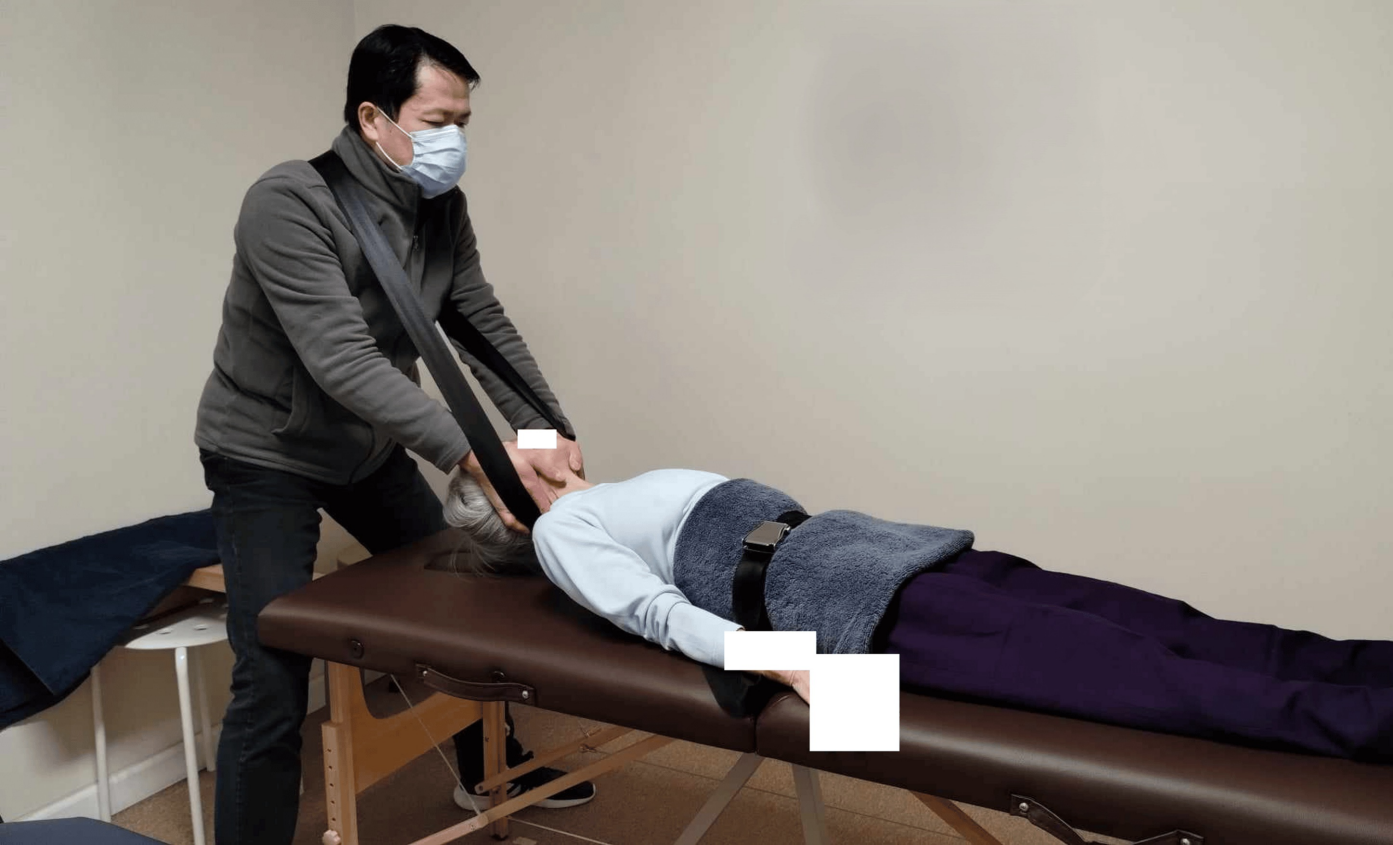
Pelvis-Stabilized Axial Spinal Traction (PSAST) Demonstration of PSAST performed on a model using a modified portable massage table. The setup includes a pelvic stabilization belt, similar in design to an airplane-style seatbelt, to secure the patient’s pelvis, and a secondary webbing strap positioned across the practitioner’s upper back to support the patient’s head and allow controlled application of traction along the craniospinal axis. The treatment table is reinforced with a 2 × 4-inch lumber extension beneath its base, positioned so that the adjacent wall provides a fixed counterforce to prevent headward displacement during traction. Image credit: Author (as practitioner) and consenting model.

The technique has been used routinely in the author’s musculoskeletal practice for over eight years, providing substantial real-world experience without observed persistent or treatment-related adverse effects. Occasional transient cervical soreness has been reported but resolves spontaneously without intervention. Although this practice-based experience does not constitute a formal safety evaluation, it supports the feasibility of further systematic investigation while acknowledging that dedicated safety studies remain necessary to establish risk profiles across broader populations (Figure 1).

Conceptual extension: self-generated axial spinal traction

Conventional spinal motions such as flexion, extension, lateral bending, and rotation primarily involve angular displacement around segmental axes. Axial elongation, in contrast, represents a longitudinal cranio-caudal tensile motion along the spine's vertical axis. This direction of loading is mechanically distinct because the spine in everyday life is predominantly subjected to gravitational and muscular compression, and meaningful decompression cannot be generated without external assistance.

A self-generated form of axial spinal elongation has been performed daily by the author for the past eight years as part of ongoing personal biomechanical observation and to explore potential preventive-care applications. This maneuver uses body positioning and partial body weight to create brief cranio-caudal elongation, generating traction forces and loading durations similar to those used in practitioner-applied PSAST. These observations are subjective and anecdotal, have not been systematically measured, and cannot be generalized to others.

This self-generated approach has not undergone formal evaluation for safety, biomechanical accuracy, reproducibility, or physiological effects, and is not presented as a clinical recommendation. Its inclusion serves only to acknowledge that axial elongation as PSAST can, in principle, be produced without an external practitioner.

Biomechanical basis for modulating cerebrospinal and glymphatic circulation

Although neurodegenerative diseases present with diverse clinical phenotypes, many share impaired glymphatic clearance as a convergent upstream mechanism [1-3]. In genetically mediated disorders such as Huntington’s disease and various spinocerebellar ataxias, polyglutamine expansion is the primary etiologic driver [4,5]. However, the efficiency of metabolic and protein-waste clearance may modulate the rate at which toxic aggregates accumulate. More effective clearance pathways, including glymphatic function, may therefore influence the timing of clinical manifestations, even though they cannot alter the underlying genetic cause.

This framework provides a conceptual rationale for exploring whether controlled cranio-caudal traction might influence CSF hydrodynamics or glymphatic transport. Established biomechanical principles support a theoretical basis by which PSAST could modulate CSF pressure gradients or spinal-canal compliance and thereby potentially influence glymphatic circulation. Direct experimental confirmation is currently lacking and will require systematic investigation.

The craniospinal compartment consists of the brain and spinal cord enclosed within the dura mater and surrounded by CSF. The intracranial dura mater is continuous with the spinal dural sac, forming a unified fluid-containing system described in standard anatomical texts. The spinal dura mater is firmly anchored near the foramen magnum superiorly and to the sacrum inferiorly, while the segment between these points retains limited longitudinal distensibility. The idea that a sufficiently strong cranio-caudal traction force could produce brief axial elongation of the spine and corresponding tensile deformation of the dural sac arises from logical biomechanical reasoning rather than from prior experimental research. Existing traction studies generally examine low-force, long-duration methods and do not explore short, higher-force pulses. This creates an unstudied space in which the effects of brief high-intensity traction loading on craniospinal mechanics remain unknown and potentially represent a worthwhile research direction. When such high forces are applied during PSAST, the vertebral column appears to undergo controlled elongation, and the dural sac appears to experience proportional tensile loading. Even small changes in dural-sac length may influence the local volume-pressure relationship within the spinal subarachnoid space, consistent with established principles of CSF anatomy and physiology.

This transient elongation is hypothesized to produce momentary CSF pressure gradients along the craniospinal axis. During traction, the slight increase in spinal subarachnoid space volume may create a relative reduction in local CSF pressure compared with the intracranial compartment, followed by re-equilibration upon release. Such brief volume-pressure oscillations could, in concept, drive bidirectional flow across the foramen magnum. Supporting this possibility, cine phase-contrast MRI studies show that even small cranial or cervical movements, including simple head rotation or nodding, generate measurable cranio-caudal CSF displacement at the craniocervical junction [7,8]. These findings suggest that CSF flow is highly sensitive to mechanical inputs produced during everyday movement. By extension, a stronger mechanical input such as PSAST could plausibly induce a more substantial CSF perturbation than ordinary head motion, although this remains untested.

**Figure 2:**
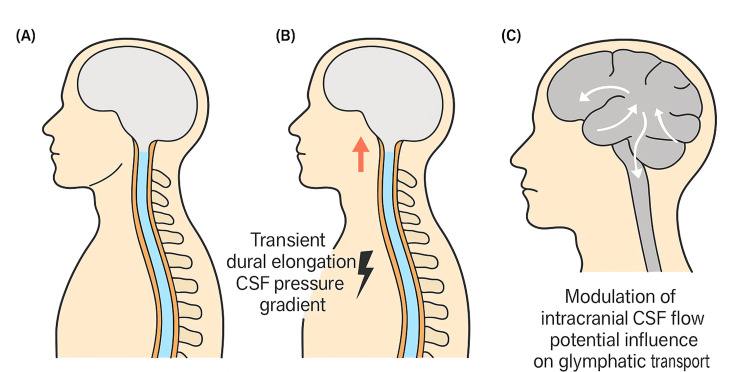
Proposed Single-Cycle Dural Pull–Recoil Mechanism During Pelvis-Stabilized Axial Spinal Traction (PSAST) (A) The craniospinal compartment, enclosed by the intracranial dura mater and its continuation as the spinal dural sac, functions as a continuous hydraulic system that permits cerebrospinal fluid (CSF) movement across the foramen magnum. (B) PSAST produces transient cranio-caudal elongation that lengthens the vertebral column and tensions the spinal dural sac along the length of the spinal canal, potentially generating momentary CSF pressure gradients within the spinal compartment. (C) During traction and subsequent release, these transient pressure gradients may propagate across the foramen magnum, generating a single-cycle dural pull–recoil stroke of CSF motion that could modulate intracranial CSF exchange and glymphatic transport. This mechanism is theoretical and requires experimental validation. Created by the author using an AI-assisted illustration tool (ChatGPT).

Because glymphatic transport depends on pressure gradients, vascular pulsatility, and unobstructed perivascular pathways [1-3], even modest enhancements in CSF mobility could influence solute movement and clearance. By combining transient CSF pressure modulation with temporary increases in dural-sac tensile loading, PSAST may provide a mechanical stimulus that supports glymphatic transport in a physiological yet previously unexplored manner. This theoretical framework does not imply therapeutic efficacy but offers a biomechanically plausible rationale for systematic investigation (Figure 2).

## Discussion

Safety of PSAST

The vertebral arteries are a primary focus of safety concern in cervical spine manipulation because they ascend through the transverse foramina from approximately C6 to C1 and curve posteriorly around the atlas before entering the cranial cavity. High-velocity, low-amplitude (HVLA) cervical manipulative procedures involving rapid rotation or combined rotation and extension have been temporally associated with vertebral artery dissection in case reports, as these movements can impose abrupt tensile, torsional, and bending loads on the arterial wall [9,10].

In contrast, controlled axial spinal elongation produces predominantly cranio-caudal decompression rather than transverse or rotational motion at the spinal segments. During such elongation, the vertebral arteries would be expected to glide longitudinally within the transverse foramina, a pattern of movement that is biomechanically less likely to generate torsional or shear stress than HVLA rotational maneuvers. This description represents the author’s biomechanical interpretation based on established anatomical relationships and is not derived from published comparative studies. This remains an inferred biomechanical advantage rather than a demonstrated clinical one. Systematic studies comparing vertebral artery wall strain or vascular adverse-event rates between axial traction and rotational manipulation are not yet available, so any differences in vertebrobasilar risk associated with PSAST should be regarded as theoretical at this stage.

Cadaveric biomechanical studies demonstrate that the cervical spine possesses substantial tolerance to axial tensile loading. Ligamentous cervical motion segments fail at approximately 1,800 N in the lower cervical spine and 2,400 N in the upper cervical region, while computational whole-neck models predict tolerances up to 4,200 N when cervical musculature is engaged [11]. Axial-tension experiments on intact human preparations further report mean failure loads of 1,550 N for skull-T3 specimens and 3,370 N for fully intact, unembalmed cadavers [12].

In comparison, PSAST applies controlled cranio-caudal traction forces equivalent to approximately 50-80% of body weight, typically not exceeding about 800 N, which represents the practical upper limit that a trained practitioner can comfortably and consistently generate in the author’s clinical experience. The traction load is applied smoothly, with tension increasing from baseline to the target force over roughly two seconds, followed by an optional brief hold of 0-3 seconds, rather than being delivered as a sudden or jerking motion. When compared with the published structural failure thresholds above, these applied forces represent safety margins of roughly twofold relative to skull-T3 preparations and approximately four- to fivefold when cervical and thoracic musculature is preserved. These comparisons provide a biomechanically reasonable, practice-based indication that PSAST traction forces operate well below known tensile failure limits; however, such inferences do not replace the need for physiological or clinical safety evaluation.

Clinical experience suggests that many patients report subjective improvement after PSAST. However, individuals with markedly rigid or ankylosed spinal segments may have limited tolerance for traction, even at low loads. Structural conditions such as ossification of the posterior longitudinal ligament (OPLL) can restrict spinal mobility and may reduce both the safety and the potential effectiveness of traction-based interventions. Patients with advanced OPLL typically require surgical assessment, and traction is often contraindicated in this setting. Careful clinical screening, including appropriate medical imaging, is recommended before initiating traction in individuals with suspected spinal canal stenosis or ligamentous ossification. OPLL has a reported prevalence of approximately 1.9-4.3% in Japan and lower rates in other East Asian populations, while it is uncommon in Western countries, where estimated prevalence ranges from 0.01 to 1.7% [13].

Clinical observations supporting the hypothesis

Clinical observations from the author’s practice suggest that PSAST may influence symptoms associated with disturbed sleep and altered craniospinal biomechanics. Among individuals receiving PSAST, improved sleep quality has been the most consistent patient-reported outcome. Many patients report deeper, less interrupted sleep following traction sessions, often independent of changes in musculoskeletal discomfort. These reports represent the author's practice-based clinical observations and have not yet been evaluated in controlled or systematic studies.

A similar pattern was documented in the patient with probable bulbar-onset ALS previously reported by the author. Prior to initiating PSAST, she experienced severe insomnia for more than one year and was unable to sustain restorative sleep. Within two weeks of starting PSAST, she reported marked improvement in sleep continuity and depth. This early change was followed by preservation of bulbar and respiratory function for 21 months, as detailed in the published case report [6], and these functional preservations have persisted through 24 months of ongoing follow-up at the time of writing.

Because glymphatic activity is greatest during deep sleep, improvements in sleep quality may reflect more effective CSF-interstitial fluid exchange. Conversely, disrupted sleep architecture, such as reduced slow-wave sleep or increased fragmentation, is associated with diminished glymphatic clearance, indicating a reciprocal relationship between sleep integrity and brain-waste removal [1-3]. The mechanism underlying reports of improved sleep after PSAST remains uncertain, but one possibility is that transient cranio-caudal decompression may influence craniospinal compliance or CSF mobility in ways that promote restorative sleep. Any potential effect on glymphatic physiology is speculative and requires formal experimental evaluation.

Although these observations are anecdotal, the recurrent pattern of sleep enhancement, together with the prolonged preservation of bulbar and respiratory function in the published ALS case [6], provides preliminary, hypothesis-generating support for further investigation of PSAST as a potential biomechanical modulator of cerebrospinal and glymphatic circulation. PSAST has not yet been demonstrated to alter CSF or glymphatic flow in vivo.

ALS as a model for hypothesis exploration

PSAST is hypothesized to influence CSF dynamics and glymphatic circulation through controlled cranio-caudal elongation of the craniospinal axis. Because many neurodegenerative disorders share impaired protein clearance and reduced glymphatic function as contributing mechanisms [1-5], any future demonstration that PSAST can modulate CSF hydrodynamics or glymphatic transport could hold exploratory relevance across a broader range of conditions.

ALS provides a practical clinical model for examining this hypothesis. The disease typically follows a relatively rapid and predictable course, with a median survival of approximately 20 to 48 months after symptom onset [14]. Although individual variability exists, this timeframe offers a feasible observation window for detecting potential biomechanical or physiological effects. Functional decline can be tracked using validated clinical tools such as the revised ALS Functional Rating Scale (ALSFRS-R), which quantifies progression across bulbar, limb, and respiratory domains [15].

Given the limited efficacy of current disease-modifying therapies for ALS [16], even modest influences on CSF movement, glymphatic clearance, or related waste-removal pathways could, in principle, produce biologically meaningful downstream effects. As a noninvasive biomechanical maneuver performed under professional supervision, PSAST offers a plausible exploratory adjunct for investigating the relationship among spinal biomechanics, CSF hydrodynamics, glymphatic function, and functional outcomes in ALS.

Contraindications and precautions

PSAST should not be performed in individuals with cervical spine instability, vertebrobasilar insufficiency, cranial or spinal vascular malformations, intracranial mass lesions or elevated intracranial pressure, severe osteoporosis, acute or suspected vertebral fractures, recent spinal or cranial surgery, active spinal or meningeal infections, malignancy involving the spine, severe spinal canal stenosis, or any condition that compromises spinal, neural, or vascular integrity.

PSAST should be used with caution in individuals with mild to moderate degenerative spine disease, a history of cervical trauma including whiplash injury, migraine or vestibular sensitivity, cervicogenic dizziness, generalized joint hypermobility or connective tissue laxity, cardiovascular or respiratory disorders that may be sensitive to transient cerebrospinal fluid pressure changes, anxiety or low tolerance to manual procedures, or in individuals who have difficulty communicating discomfort or neurological symptoms. The procedure should be stopped immediately if the patient develops new or worsening neurological symptoms, severe headache, dizziness, visual changes, or significant neck pain.

Disclaimer

PSAST is an exploratory, investigational procedure intended for controlled application only under the supervision of a qualified professional. Individuals must be thoroughly evaluated and screened for all contraindications and precautions described before undergoing PSAST.

The author disclaims all responsibility and liability for injury, harm, or adverse outcomes resulting from unsupervised use, improper application, deviation from the described technique, or use in individuals for whom PSAST is contraindicated.

This technical report does not establish therapeutic efficacy for PSAST. Further biomechanical studies, advanced imaging, and controlled clinical research are necessary to assess safety, characterize physiological effects and determine any potential applicability in neurodegenerative or other clinical conditions.

## Conclusions

PSAST is designed to generate brief, controlled cranio-caudal elongation of the vertebral column and spinal dural sac. When applied within physiological limits and with appropriate precautions, the technique appears to have a wide biomechanical safety margin. Although direct physiological evidence is not yet available, the hypothesis that transient modulation of craniospinal compliance and CSF pressure gradients may influence CSF movement and glymphatic transport provides a coherent framework for further investigation. Patient-reported improvements in sleep quality are noteworthy, given the strong relationship between restorative sleep and glymphatic function, although such observations remain preliminary and practice-based.

Neurodegenerative diseases characterized by impaired protein clearance may offer practical early models for hypothesis testing, with ALS being particularly suitable because of its predictable functional trajectory, validated assessment tools, and relatively short clinical timeframe. Future work should include biomechanical studies, advanced imaging, and controlled clinical research to determine whether the hypothesized effects of PSAST on CSF hydrodynamics, sleep architecture, or glymphatic physiology are reproducible in vivo. Because CSF participates not only in metabolic waste clearance but also in the distribution of hormones, neuromodulators, and other biologically active solutes, further research may examine whether mechanically influenced CSF dynamics affect these pathways. At this stage, PSAST represents a conceptual and technical proposal for investigation, and no therapeutic claims can be made until supportive physiological and clinical evidence becomes available.
